# Supplementary material for: Synergistic Activity of Pep16, a Promising New Antibacterial Pseudopeptide against Multidrug-Resistant Organisms, in Combination with Colistin against Multidrug-Resistant Escherichia coli, In Vitro and in a Murine Peritonitis Model
Source: Antibiotics (Basel). 2023 Jan 3;12(1):81. doi: 10.3390/antibiotics12010081 (PMC9854584; doi:10.3390/antibiotics12010081)
Supplement: Supplementary file 1 [file antibiotics-12-00081-s001.zip › antibiotics-2111717-Supplementary Figure S1.pdf]

Supporting Information

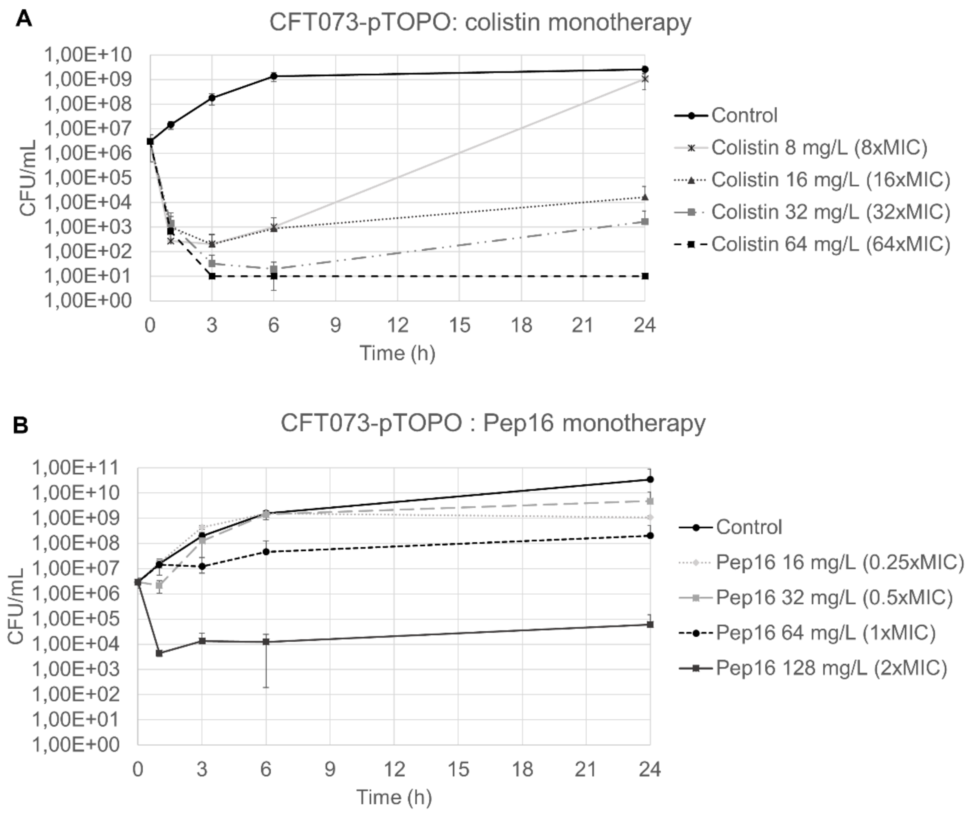

Figure S1. Time–kill curves at high concentrations of Pep16 or colistin monotherapy on CFT073-pTOPO. **(A)** Pep16. **(B)** Colistin. Data expressed as the mean and the standard deviation. All experiments were conducted at least 3 times. A bactericidal effect is defined as a 3 $\log_{10}$ decrease in CFU counts compared with the initial inoculum.
